# Cultivation-success of rare soil bacteria is not influenced by incubation time and growth medium

**DOI:** 10.1371/journal.pone.0210073

**Published:** 2019-01-10

**Authors:** Viola Kurm, Wim H. van der Putten, W. H. Gera Hol

**Affiliations:** 1 Department of Terrestrial Ecology, Netherlands Institute of Ecology (NIOO-KNAW), Wageningen, The Netherlands; 2 Laboratory of Nematology, Wageningen University, Wageningen, The Netherlands; Wageningen University, NETHERLANDS

## Abstract

Rare bacterial species have recently attracted interest due to their many potential beneficial functions. However, only little is known about their cultivability. In this study we test the hypotheses that the use of flow cell-sorting for cultivation results in a high proportion of rare soil bacterial isolates relative to bacterial taxa that are abundant in soil. Moreover, we investigate whether different oligotrophic cultivation media and a prolonged incubation time increase the number of cultivated rare species. In a cultivation study we used flow cell sorting to select for small cells and to separate single cells, and grew bacteria on different oligotrophic media with prolonged incubation times. The abundance of the isolates in the field was assessed by comparing them to a 454-sequencing dataset from the same soil. Consequentially, all bacterial isolates were classified as either rare (<0.01% relative abundance) or abundant (>0.01% relative abundance) in the field soil. We found more bacterial taxa among the isolates that were abundant in soil than would be expected by the proportion of abundant species in the field. Neither incubation time nor growth medium had an influence on the recovery of rare species. However, we did find differences in time until visible growth on the plate between different phylogenetic classes of the isolates. These results indicate that rare cultivable species are active and not more likely to be dormant than abundant species, as has been suggested as a reason for their rarity. Moreover, future studies should be aware of the influence incubation time might have on the phylogenetic composition of the isolate collection.

## Introduction

The invention and improvement of high-throughput sequencing techniques has led to the discovery of a large proportion of previously undetected rare species in bacterial communities and multiple studies indicated the importance of rare soil bacterial species for various ecosystem functions [1, 2] (rare species we define here as bacterial OTUs occurring at a relative abundance of <0.01% in the community [3]). However, sequencing can merely show which species are present at a specific time point. It provides useful information about the potential functions of a particular species or a community, but these still have to be tested in experimentally to verify actual functioning. Also species traits that might be related to causes of species rarity, such as slow growth rates, the sensitivity to environmental conditions, competition or predation, have mostly been studied by analysing general changes in community composition or by other molecular techniques, such as stable isotope probing [4, 5]. These potential traits will have to be verified *in vitro*. Therefore, cultivation is still required to study bacterial species in isolation and confirm potential traits. Present cultivation approaches are still largely untargeted without prior knowledge on the cultivable species except for the use of cultivation media selecting for or excluding a narrow range of species [6]. Hence, there is a clear need for more informed cultivation approaches to increase the chances of capturing bacteria of interest for subsequent studies both with respect to species abundance and to other traits, such as phylogeny.

It has frequently been demonstrated that only a minority of all bacterial species can be cultivated; this has been named ‘the great plate count anomaly’ [7]. In addition, it is still poorly investigated how the species that can be captured by cultivation, rank in abundance in the field community from which they have been isolated. The only studies that we are aware of, which looked at the abundance of cultivated species and rare species specifically, report contrasting results [8, 9] that could partly be due to differences in cultivation techniques. For example, there have been few, if any, studies on how different cultivation media might affect the abundance range of species captured. However, cultivation-independent studies give valuable information about some attributes of rare species that can be used to increase cultivation success. Portillo, Leff [10] showed, by separating bacterial communities in different size classes, that low abundant species were mostly found in the small-size fraction of <0.8 μm. Therefore, the selection of small cells might increase the proportion of cultured rare species. In addition, it has been suggested that rare species might be low abundant in the community due to a poor competitive ability compared to species that are more abundant [11]. Therefore, the separation of single cells could favour the growth of rare species as it prevents direct competition.

In addition, some species that are rare in the field might belong to a temporally inactive seed-bank with dormancy as a life-history strategy to cope with unfavourable conditions [12]. Dormancy could lead to a poor cultivability of rare species in short-term cultivation approaches. While a large proportion of dormant low abundant bacterial species has been shown to have the ability to become highly active and increase in abundance [13], it can be expected that re-activation from dormancy can cause these rare species to require longer incubation times for visible growth in cultivation approaches. Moreover, rare species have often been assumed to be slow growing, which would also require a prolonged incubation time to increase the proportion of cultivated rare species. However, in a follow-up study on bacterial isolates we did not find a difference in growth rate between rare or abundant species [14].

Not only is little known about the influence of cultivation media and incubation time on the cultivability of rare bacterial species, but also on species phylogeny. Extended incubation times have been indicated to lead to an increased cultivation of rarely isolated groups, such as Acidobacteria, Gemmatimonadetes or Planctomycetes [15]. However, it has seldom been systematically tested if phylogeny is related to incubation time. Growth medium might also influence the phylogenetic composition of bacterial isolates. It has been suggested that nutrient-rich media can inhibit the growth of obligate slow-growing oligotrophic species [16]. Especially members of the Alphaproteobacteria have been indicated to grow preferentially on nutrient-poor media [17]. Moreover it has been suggested that autoclaving agar-containing media with high phosphate concentrations might inhibit the growth of some bacterial classes [18, 19]. Examples of media that have been recommended for the isolation of soil bacteria are dilute tryptone soy broth or dilute nutrient broth (DNB) [20, 21]. Different gelling agents, such as gellan gum, instead of agar, have been proven effective as well [20]. Attempts to mimic soil conditions have also led to the development of soil extract media, which yielded strains that could not grow on conventional media before [22, 23]. Although these media have been used to successfully isolate as yet uncultivated species, remarkably little is known about the effect of different cultivation media on the general phylogeny of the isolated species.

The aim of the present study was to determine the relative abundance of cultivated bacterial species in field soil. We focused on the cultivation of bacterial species that are low abundant in soil, in order to enable further tests of their traits experimentally, e.g. as Kurm, van der Putten [14], using flow cell sorting to select small bacterial cells. Furthermore, we investigated whether long incubation times and different cultivation media would influence the proportion of cultivated rare species or the phylogenetic composition of the set of isolates. We found several rare species among our isolates, but at a lower proportion than would be expected from the proportion of rare bacterial species in the soil bacterial community. Neither incubation time nor medium had an influence on the abundance of cultivated species, whereas incubation time influenced phylogenetic composition.

## Material and methods

### Isolation of bacteria

In May and June 2014, soil samples for bacterial cultivation were taken from a long-term biodiversity experiment, named CLUE, near Ede (Gelderland, the Netherlands, 52°04′N 05°45′E) [24] using a soil corer with a diameter of 3 cm at a depth of 0–20 cm. The long-term biodiversity experiment has been installed in 1996 on an ex-arable field, abandoned from agriculture in 1995. The soil type is sandy loam soil with the following chemical characteristics: Olsen *P* = 90 ± 3 mg/kg, %orgC = 4.2 ± 0.3, C:N = 16.8 ± 0.1, Min-N = 10.6 ± 0.8 mg/kg, pH = 6.2 ± 0.1 [25]. Bacteria were isolated by a flow sorting in combination with different media. The following cultivation media were used: 0.1 strength tryptone soy agar (0.1TSA; 3 g.l^-1^ tryptone soy broth, 15 g.l^-1^ bacto agar), 0.01 strength TSA (0.01TSA; 0.3 g.l^-1^ tryptone soy broth, 15 g.l^-1^ bacto agar), dilute nutrient broth agar (DNB; 0.03 g.l^-1^ meat extract, 0.05 g.l^-1^ peptone, 8 g.l^-1^ gellan gum, 1.26mmol CaCl_2_), water yeast agar (WYA; 5 g.l^-1^ NaCl, 0.05 g.l^-1^ yeast extract, 20 g.l^-1^ bacto agar) and soil agar with and without the addition of nutrients (SA+ and SA- respectively, see Kurm et al. (2017)). Five 96-well plates were prepared from each medium. One day prior to cell sorting 5.4 g of sieved soil were added to 130 ml phosphate buffer and shaken for 1.5 h at 120 rpm. Sonication was performed 2x 1 min to detach bacterial cells from soil particles. After shaking again for 0.5 h the soil solution was passed through a 45 μm sieve. 130 ml of phosphate without added soil was used as a control. The soil solution and the control were stained with SYBR green stain (Sigma-Aldrich, St. Louis, Missouri, USA). Using a flow cytometer (MoFlo Legacy Cell Sorter; Beckman Coulter, Miami, Florida, USA) SYBR-Green stained particles of an approximate size of 0.5 μm were sorted into single wells of the 96-well plates. This method is known as fluorescence activated cell sorting (FACS) [26]. The size-selection was made to enhance the proportion of bacteria that are rare in field soil as it has been shown previously that the small-sized fraction of cells contains on average more rare species [10]. The sorted 2880 particles represented approximated 0.35% of all particles (stained and unstained) in the sample. The plates were inspected every other day by eye for visible bacterial growth for 5 months. Upon visible growth bacteria were subcultured by transferring them individually to fresh 0.1TSA agar on regular plastic petri-dishes. This study was conducted before our follow-up study showed that there is no difference between rare and abundant taxa with respect to growth rates [14]. Therefore, rare species were still expected to require longer incubation times. We define cultivation success as the number of isolates that could be retrieved relative to the number of wells that were inoculated.

### Identification of bacterial cultures

The 16S rRNA was amplified with colony PCR using bacterial cells picked from bacterial isolates grown on 0.1TSA plates, suspended in SDS-lysis buffer and lysed for 5 min at 95°C. DNA from isolates recalcitrant to this method was extracted using the ZR fungal/bacterial DNA MiniPrep kit (Zymo Research, Irvine, U.S.A.), the Power soil DNA isolation kit (MO BIO laboratories, Carlsbad, U.S.A.) or the QIAmp DNA Mini kit (Qiagen, Venlo, The Netherlands) and 1 μL DNA was used as a template in the PCR. 16S rDNA fragments were amplified using the primers pA (5’-AGAGTTTGATCCTGGCTCAG-3’) and 1492r (5’-GRTACCTTGTTACGACTT-3’) and sequenced by Sanger sequencing by Macrogen (Amsterdam, The Netherlands) or Baseclear (Leiden, The Netherlands) using the primer 515f (5′-GTGCCAGCMGCCGCGGTAA-3′). After sequence quality trimming using the program Phred [27], sequences of a minimum length of 83 and a maximum length of 541 bases were blasted against the greengenes and SILVA database using the SINA alignment service for phylogenetic identification [28] with a k-mer length of 10 bases. The sequences were deposited in Genbank under the accession numbers KX503324-KX503369.

### Estimation of relative abundance in field soil

For estimation of abundance in field soil the isolate sequences were blasted against an OTU reference table using NCBI blastn (NCBI, Bethesda, Maryland, USA) [29]. The OTU reference table had been generated from a 454-sequencing database containing sequences from seven soil replicates that were collected from the same site as the isolates, with 20000–50000 reads per sample [30]. The isolate sequences were matched to an OTU with a percentage identity cut-off of 97%. The mean relative abundance of the OTU match in the sequencing database was taken as the relative abundance of the isolate in soil. Following our earlier definition, rare OTUs are those occurring at a relative abundance <0.01% and abundant OTUs as those occurring >0.01% in the sequencing database.

For a detailed description of isolation of soil bacteria, identification of isolates, and estimation of relative abundance in the environment see [14]. The present study contains a subset of the isolates used in [14] as additional isolates were obtained by other methods that could not be directly compared with the flow sorting.

### Data analysis

All statistical analyses were performed in R studio with R version 3.2.3. [31].

Abundance distributions between the field soil and unique isolated OTUs were compared with a Kolmogorov Smirnov-test. The abundance distributions of the different phylogenetic classes were tested in the same way. For this purpose, OTUs that were not detected in the field community, but present in the whole sequencing dataset were assigned an abundance of 1*10^−7^. The effect of different cultivation media on both the total number of isolates and the number of members of the different classes was tested with a generalized linear model using the glm() function, with the distribution family specified as “poisson” (n = 5). The overall significance of the models were determined with the Anova function from the car package [32] and pairwise comparisons were performed with the function lsmeans from the package lsmeans [33]. The proportions of the five different classes found in this study was compared to their proportions in field soil (taking into account only the number of OTUs within a class, irrespective of their abundance) using a Chi-square test. The effect of cultivation medium on the square-root-transformed relative abundance in the environment averaged over the five replicates was tested with a one-way anova (n = 5). The relationship between time until visible growth on plate and relative abundance was tested with a Spearman rank correlation test. Similarly, the relationships between average growth rate (as assessed in Kurm, van der Putten [14]) and time until visible growth on the plate, and between relative abundance and the number of times the same OTU was isolated, were determined. Whether different classes differed in their average time until visible growth was assessed with a linear model, using the average time until visible growth for each unique OTU as the dependent variable. To detect effects of the different growth media on time until visible growth a linear model was used with time until visible growth averaged over the five replicates per medium. Accumulation curves were generated for the time until visible growth on the different media and differences between the curves were tested with a Kolmogorov-Smirnov test.

## Results

Bacterial growth was observed in 178 wells. Of these 178 growth events 172 isolates could be subcultured, corresponding to a cultivation success of 6% (defined as the number of isolates retrieved relative to the number of well that were inoculated). Of these isolates 113 were phylogenetically identified by sequencing and matched to an OTU from the 454-sequencing database. Based on the OTU match, the isolates could be grouped into 43 different OTUs (Table A in S1 File).

### Relative abundance

**Fig 1:**
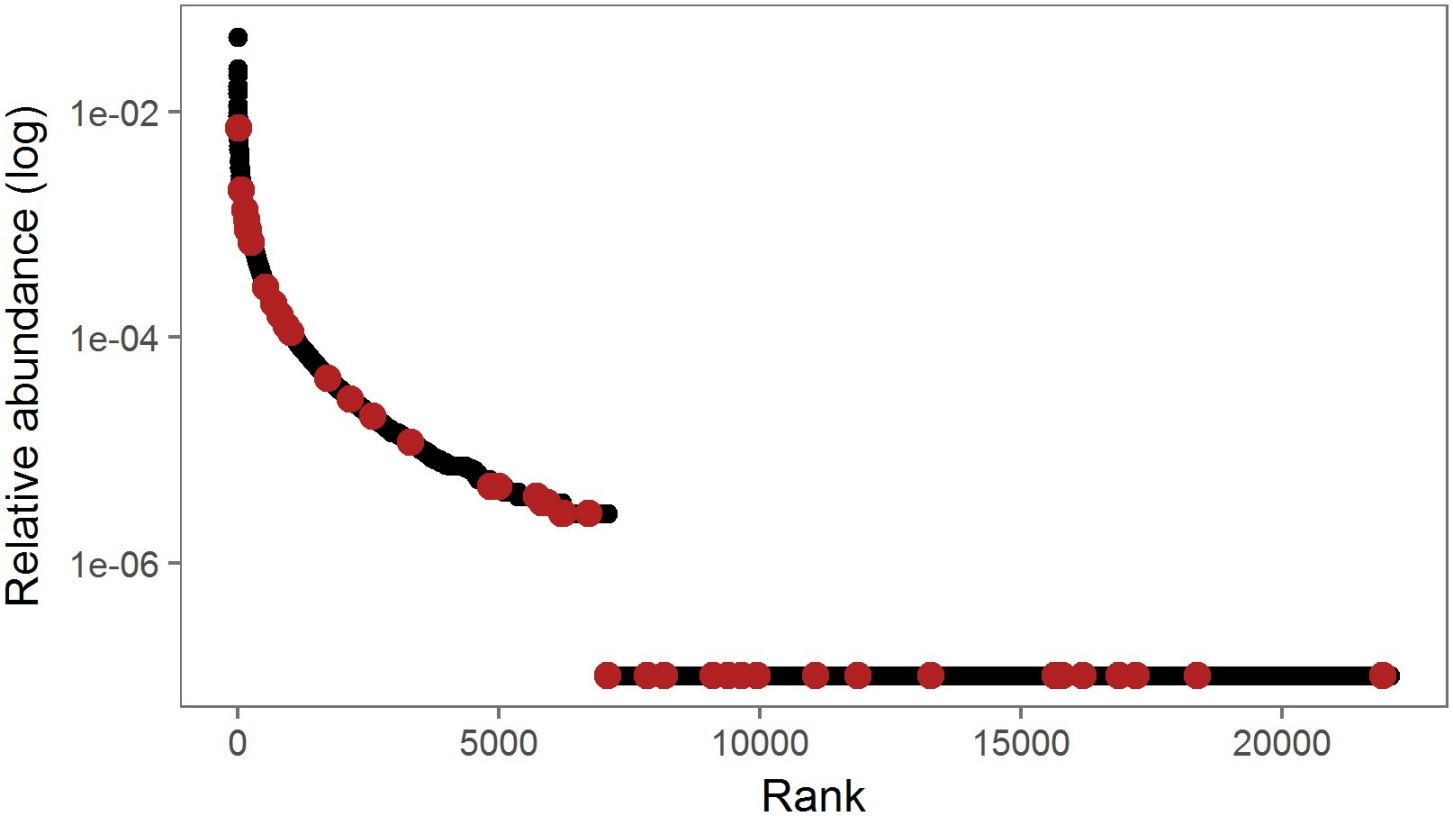
Species abundance distribution of the field community. Species abundance distribution of all OTUs in the field community (black) displayed as log-transformed relative abundance vs. species rank in the community; OTUs that were matched to a bacterial isolate are marked in red.

The species abundance distribution of the sequenced field community shows a high number of rare species, but bacterial isolates were found to rank relatively high within this abundance distribution (Fig 1). The relative abundances of the isolated OTUs differed significantly from the relative abundances of the field community (Kolmogorov-Smirnov test; D = 0.3, p<0.01). In the field community, across all OTUs, 95% of all OTUs were rare and 5% where abundant, while from the 43 isolates with unique OTUs, 30% were abundant and 70% were rare, based on their abundance in the field (Table 1).

**Table 1:** Number of isolates from the respective cultivation medium, abundance and incubation time.

	No. of isolates	0.01TSA	0.1TSA	DNB	SA-	SA+	WYA
***No. of isolates***							
*Total no. of isolates*	113	36	23	13	7	15	19
*No. of rare isolates*	76	22	14	10	5	11	14
*No. of abundant isolates*	37	14	9	3	2	4	5
*No. of strains*	43	18	17	10	7	9	11
*No. of rare strains*	30	10	9	7	5	7	10
*No. of abundant strains*	13	8	8	3	2	2	1
*No. of unique strains*	26	4	8	2	1	4	7
*No. of shared strains*	17	14	9	8	6	5	4
***Class***							
*No. Actinobacteria*	22	6	4	7	1	1	3
*No. Bacilli*	11	4	1	0	1	4	1
*No. Alphaproteobacteria*	28	10	5	1	2	2	8
*No. Betaproteobacteria*	17	6	5	2	1	1	2
*No. Gammaproteobacteria*	35	10	8	3	2	7	5
***Incubation time***							
*Average incubation time*	38.74	42.06	19.89	31.38	60.50	31.85	55.94
*SE incubation time*	2.73	4.89	4.52	6.22	14.69	5.35	6.42

Cultivation success of bacterial isolates differed significantly between the media (Anova, Χ^2^ = 33.15, p<0.01). Most isolates were obtained from 0.01-strength and 0.1-strength tryptone soy broth agar (0.01TSA and 0.1TSA), whereas least isolates grew on soil agar without added nutrients (SA-) and dilute nutrient broth agar (DNB) (Figure A in S1 File, Table 1). Of the 43 unique OTUs, 60% were specific to one medium. Most of these unique OTUs were obtained from 0.1TSA and WYA (8 and 7 strains respectively). The relative abundances of isolates growing on one medium were highly variable. There was no significant difference in average field abundance of isolates between the different cultivation media as bacterial isolates that were identified as rare or abundant in the field could be obtained from all media (anova, F_5,22_ = 1.5, p = 0.23; Figure B in S1 File).

The first growth event of a bacterial isolate on the 96-well plate was observed 3 days after inoculation, the last after 133 days. On average isolates grew after 39 (± 29) days (Table 1). OTUs that were isolated several times were obtained at the beginning, as well as at the end of the experiment. Isolates that were only retrieved once in this experiment were found at the beginning, but also after approximately 3 months of incubation. There was no significant correlation between the relative abundance of the bacterial isolates in the field and time until visible growth on plate (Spearman correlation, rho = -0.01, p = 0.91). The growth medium did not significantly influence the time until visible growth on the plate of the bacterial isolates (Anova, F_5,22_ = 1.93, p = 0.13). The first bacterial growth event was observed after 3 days on SA+ medium and the last after 133 days on SA- and 0.01TSA, but on each medium bacterial growth events were widely distributed over time leading to no significant differences. However, the pattern of cumulative growth events of bacterial isolates over time differed between the cultivation media. Whereas on 0.1TSA bacterial growth occurred mostly at the beginning over a short time period and levelled off after approximately 20 days, other media such as 0.01TSA and WYA showed a second series of bacterial growth events after approximately 50 days (Figure C in S1 File). Accumulation curves of bacterial growth events differed significantly between 0.1TSA and 0.01TSA, WYA, DNB and SA- (Table B in S1 File). Most of the growth events occurred within 3 months of incubation. Thereafter, only very few new growth events were observed.

### Phylogeny

**Fig 2:**
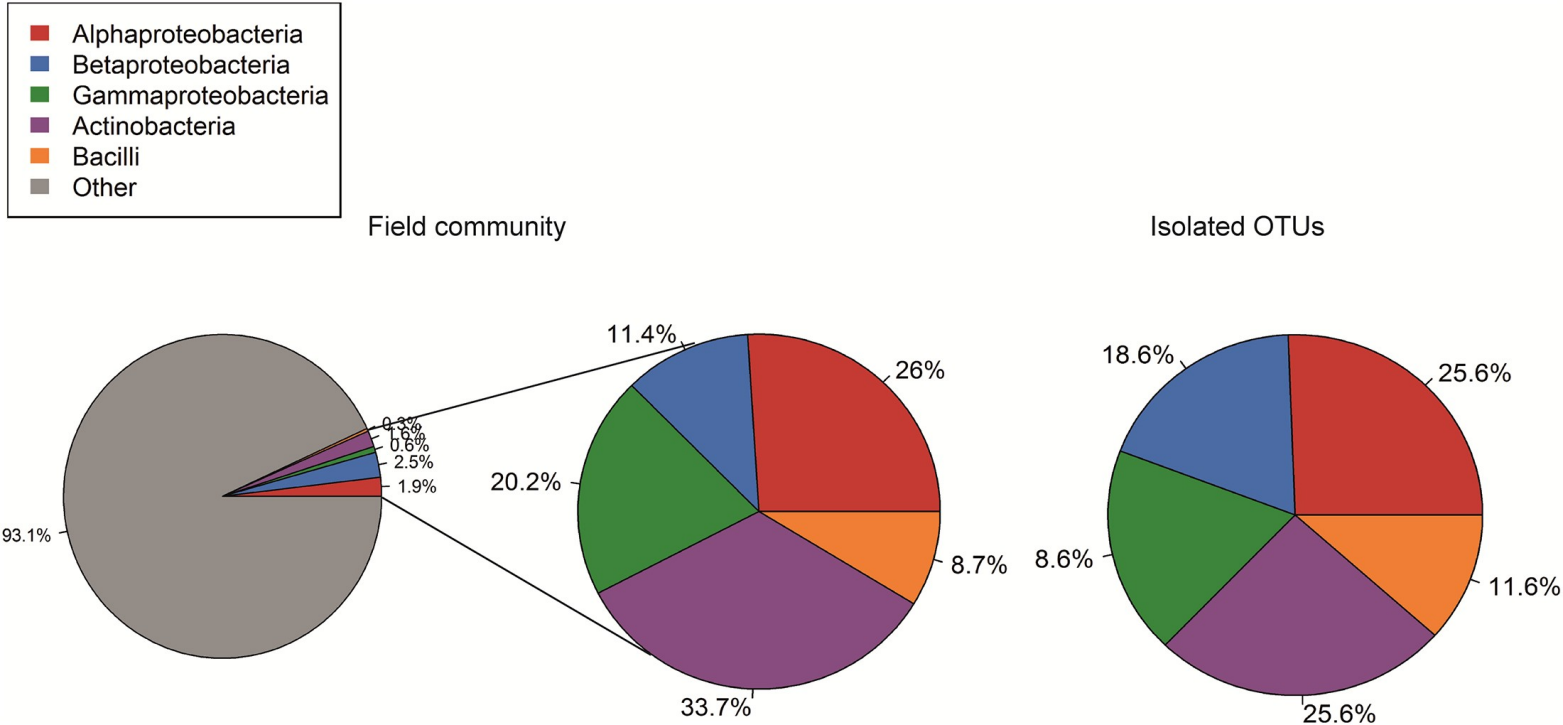
Phylogenetic composition of the bacterial field and isolated communities. Percentage of the classes Alpha-, Beta- and Gammaproteobacteria, Actinobacteria and Bacilli in the field community including all other 80 classes that were not isolated in this study as “Other” and the percentage of the 5 isolated classes only in the field community, as well as the percentages of the 5 classes in the set of isolated OTUs (n = 43).

**Fig 3:**
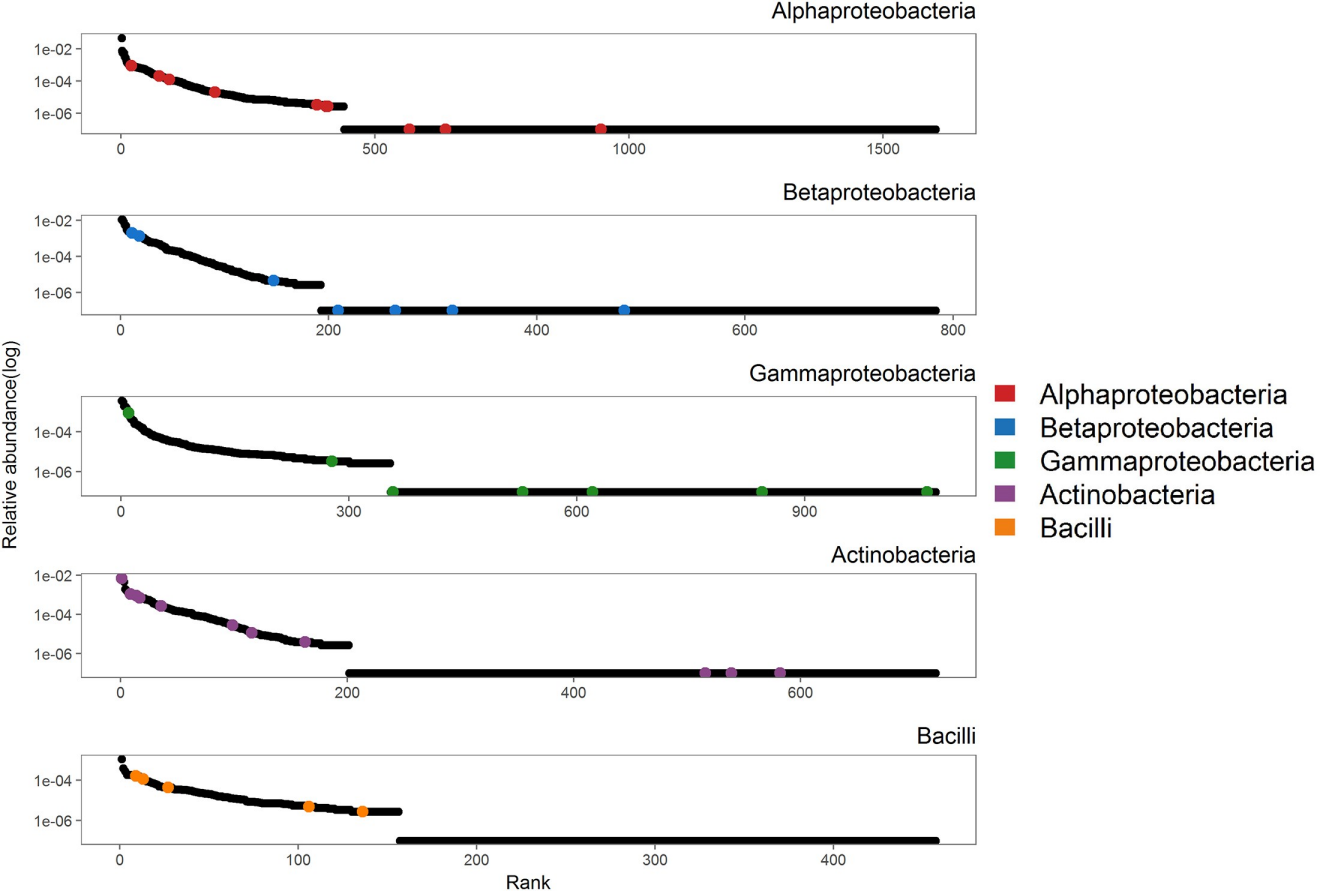
Species abundance distribution of bacterial classes. Species abundance distributions of the OTUs in the field community belonging to the 5 different classes isolated in this study displayed as log-transformed relative abundance vs. species rank in the community; OTUs that were matched to a bacterial isolate are marked in colour.

All isolates matched with a similarity of 98%-100% sequence identity to already cultivated species. The isolates belonged to 5 different classes (Actinobacteria, Bacilli, Alphaproteobacteria, Betaproteobacteria and Gammaproteobacteria), 13 orders and 23 families (Table A in S1 File). The proportions of the different classes among our isolates was similar to the proportions of the classes in the field soil from which they were isolated (Chi-square test, Χ^2^ = 10_8_, p = 0.27; Fig 2). However, there were differences in the abundance distributions of the different bacterial classes between the field community and the isolates. There were significantly more abundant and less rare species among the isolated Actinobacteria and Bacilli than would be expected based on their abundance distribution in the field, but there were no differences for the Proteobacteria (Kolmogorov-Smirnov test, Alphaproteobacteria: D = 0.4, p = 0.11, Betaproteobacteria: D = 0.2, p = 0.80, Gammaproteobacteria: D = 0.2, p = 0.99, Actinobacteria: 0.5, p<0.01, Bacilli: D = 0.7, p = 0.03, Fig 3).

All bacterial classes were found on all media, except for Bacilli, which were not found on DNB. There were no significant differences in bacterial class occurrence between the different media (Anova, Alphaproteobacteria: Χ^2^: 17.2_5_, p<0.01; Betaproteobacteria: Χ^2^: 8.8_5_, p = 0.12, Gammaproteobacteria: Χ^2^: 11.3_5_, p = 0.05; Actinobacteria: 9.2_5_, p = 0.10; Bacilli: 9.9_5_, p = 0.08; Figure D S1 File). For Alphaproteobacteria and Gammaproteobacteria the overall occurrence was significantly different, but this effect was no longer detectable in pairwise comparisons between the media.

**Fig 4:**
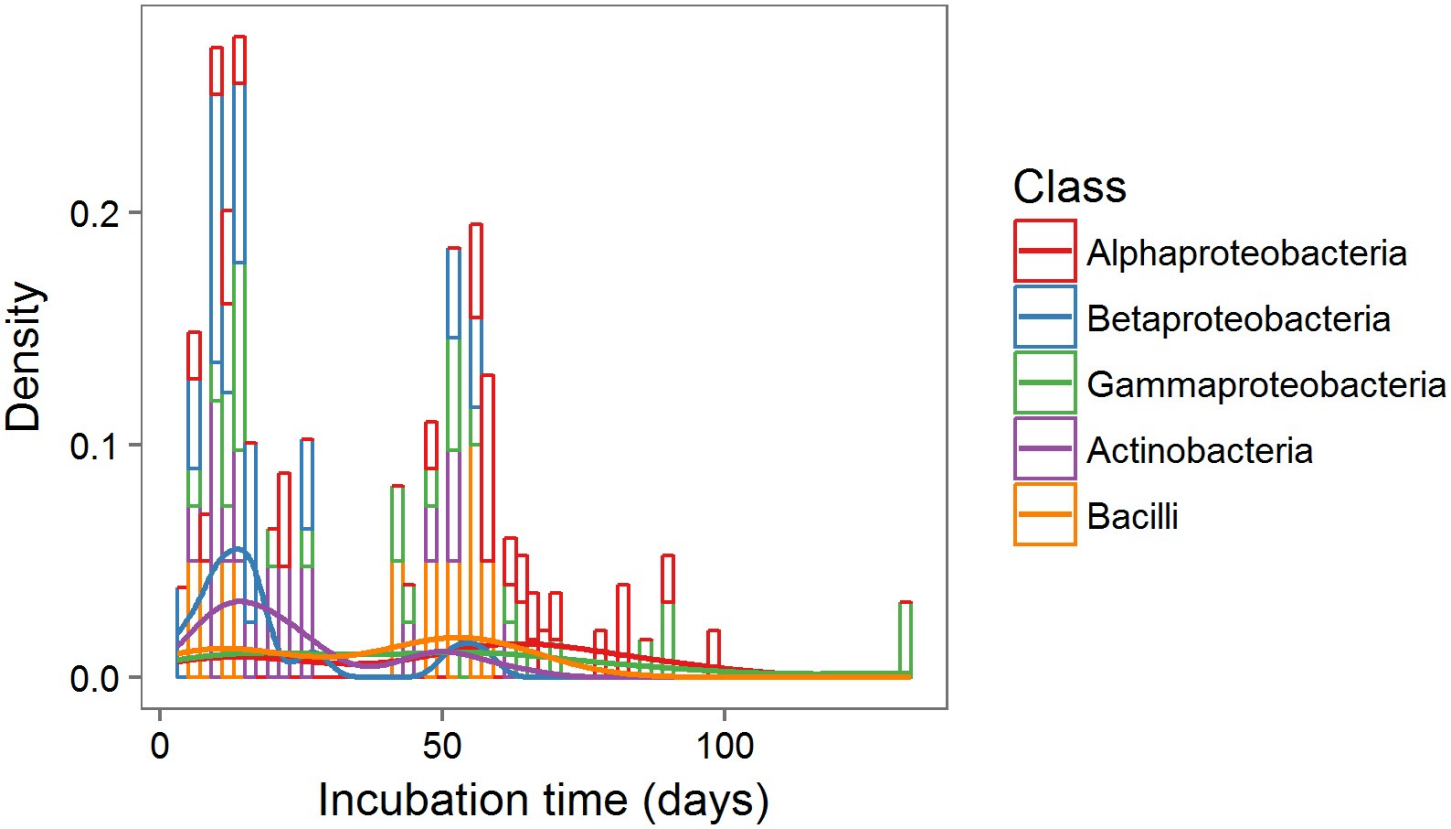
Time of first bacterial growth on the plates. Density plot of number of bacterial isolates showing visible growth on the plate along incubation time; lines represent densities of the different phylogenetic classes.

Bacterial isolates belonging to different phylogenetic classes differed significantly in their time until growth on the plates (Anova, F_4,36_ = 5.14, p<0.01). Actinobacteria and Betaproteobacteria on average were observed earlier than Gammaproteobacteria and Alphaproteobacteria (Fig 4).

## Discussion

Soil bacterial communities consist of a vast majority of rare species. While the cultivation approach presented in this study captured more rare than abundant bacterial species, the proportion of rare species among the isolates was lower than the actual proportion of rare species in the field community and proportionally more isolated species are found in the abundant part of the species abundance distribution in the field community compared to the long tail of rare species. The recovery of a high proportion of abundant species is unexpected since we selected for rare species by sorting small cells with a diameter ~0.5 μm and preventing interspecific competition. On the one hand, this can result from the naturally larger cell density of abundant species, which makes them more likely to be recovered by chance. On the other hand, species that are abundant in the field might possess traits that facilitate their growth in culture. Although the restricted sample size and cultivation effort and the exclusion of cells with a diameter >0.5 μm does not allow a direct quantitative comparison between the cultivation-independent field community and the cultivated isolates, our results indicate that cultivation might select for bacterial species with a rather high abundance rank in the field community.

As rare bacterial species were not restricted by competition, the choice of cultivation media might have favoured the growth of more abundant species. Although the media used in this study differed in composition, most of them contained easily degradable substrates and commonly support the growth of a variety of saprotrophic species. It has been suggested that rare bacterial species might be adapted to specific niches or scarce substrates accounting for their low abundance [34]. Alternatively, rare species might require chemical compounds produced by other species either as signalling compounds or as a nutrient source [35]. These species consequentially could not be cultured with common media and in isolation. Soil agar should have provided substrates that are naturally present in soil and similar media have been reported to lead to the cultivation of a diverse array of species. However, autoclaving can lead to significant changes in the structure of soil organic carbon and especially increase the amount of easily degradable carbohydrates [36]. In contrast to species abundance, the media differed in the number of cultured species. TSA medium of 0.01- strength showed the highest cultivation success indicating that this low-strength medium can be even more efficient for bacterial cultivation than the frequently used 0.1 strength TSA [21]. This might be because of the lower nutrient concentration of 0.01 strength TSA that more closely resemble oligotrophic conditions in soil. Soil agar showed a poor cultivation success, which might have been due to a relatively high phosphate concentration in the medium [18, 19]. However, media without phosphate showed equally low cultivation success. The respective cultivation media for this study were chosen because they are commonly used in many cultivation studies. In addition, they have been shown to allow for the isolation of a diverse set of phylogenetically different species [20, 37]. Future studies should consider the use of media that more closely mimic soil conditions.

In contrast to our expectations, species abundances, defined as their field abundance, showed no relationship with time until visible growth indicating that among the cultivable bacteria rare species were not more likely to be dormant than abundant species. This finding is in accordance with studies demonstrating that a large proportion of the rare part of bacterial communities is highly active [38, 39]. Moreover, a later study on the isolates captured in the present cultivation approach showed high potential growth rates for many of the rare species [14]. The high activity of rare species generates the question why these species are low abundant in the field. Possible causes include interspecific competition [11], which was excluded by flow cell sorting in this cultivation study. Also predation, for example by protists, nematodes or enchytraeids in soil, might reduce the abundance of especially fast growing bacterial species [40]. Regardless of the causes of the rarity of fast growing, active species our results show that for their cultivation long incubation times are not required. However, longer incubation times can increase the overall cultivation success for cultivation media, such as 0.01TSA and WYA indicating a merit of such long incubations for these media.

While the proportions of the classes Alpha-, Beta- and Gammaproteobacteria as well as Actinobacteria and Bacilli were remarkably similar to their proportions in soil, we found differences between the relative abundance distribution of our isolates and of these classes in the field community. Isolates belonging to the Actinobacteria and Bacilli were on average more abundant in soil than would be expected. This was especially pronounced for the Actinobacteria, although they were among the most commonly isolated classes in this experiment and hence a higher number of rare species would be expected by chance. Studies concentrating on enhancing the cultivation success of Actinobacteria have suggested that members of this class are slow growing and sensitive to competition [41]. However, this cannot explain the underrepresentation of rare Actinobacteria in the present study, as our approach excluded competition and provided long incubation times.

Surprisingly, many taxa were not present among our isolates that have previously been found with similar cultivation approaches, such as members of the phyla Acidobacteria, Verrucomicrobia and Gemmatimonadetes [37, 42]. It is possible that the flow sorting approach and prior treatment of the soil samples discriminated against these bacterial taxa, for example due to sensitivity of some taxa to sonication or to SYBR Green-staining [43, 44]. Alternatively, isolates that were cultured but that could not be identified by Sanger sequencing, either due to being recalcitrant to DNA extraction or subsequent PCR, might contain additional bacterial taxa. It has been shown that primers that were formerly thought to be universal for the bacterial 16S rRNA gene, discriminate against Verrucomicrobia, including the 1492r-primer [45] that was used in our study.

Although there was no significant differences between growth media with respect to the recovery of the different classes, we did find differences in time until visible growth as Alpha- and Gammaproteobacteria required longer incubation times until they visibly grew on the plate than Actinobacteria and Betaproteobacteria. These differences between classes suggest that bacterial growth in culture might not be completely random, but partly dependent on bacterial phylogeny. Mitsui, Gorlach [46] found that Alphaproteobacteria needed longer incubation times than other taxa and suggested slow growth and an oligotrophic life-style as the underlying mechanism. For the Gammaproteobacteria, however, we can only speculate about the possible causes of such temporal dynamics. First, members of this class might have been disproportionally inactive in the soil due to unfavourable condition at the time of sample collection. Second, the isolated species might have been less efficient in adjusting to changes in environmental conditions, such as transfer to an artificial medium. The differences between taxa with respect to time until visible growth indicates an increase in phylogenetic diversity with progressing incubation time.

In conclusion, we show that the bacterial species that could be cultivated selecting for small cells on different cultivation media represent more frequently the abundant proportion of the field community than would be expected. It is possible that also other culture collections, using similar cultivation methods, might be skewed towards the more abundant species. Hence, insights originating from the study of isolated species so far might be based on the more abundant species, which could behave differently from the rare ones. Moreover, we conclude that incubation time does not influence the number of rare species that can be cultivated. However, incubation time can change the phylogenetic composition of a culture collection. Many steps still have to be taken to comprehensively study bacterial cultivability as the present study represents only one of many available techniques.

## Supporting information

S1 File **Table A. Information on OTUs retrieved in this study.** Unique OTUs, phylogenetic affiliation, relative abundance in the field soil and the number of retrieved isolates for each OTU in the present cultivation approach. **Table B. Statistical results for differences between accumulation curves.** Results of pairwise Kolmogorov-Smirnov tests between the accumulation curves of bacterial growth events on the different cultivation media (0.01TSA = 0.01 strength tryptone soy agar, 0.1TSA = 0.1 strength tryptone soy agar, DNB = dilute nutrient broth agar, SA- = soil agar without nutrient addition, SA+ = soil agar with nutrient addition, WYA = water yeast agar). **Figure A. Cultivation success on the different media.** Cultivation success (number of isolates relative to all 96 wells inoculated) of bacterial isolates on the different cultivation media (0.01TSA = 0.01 strength tryptone soy agar, 0.1TSA = 0.1 strength tryptone soy agar, DNB = dilute nutrient broth agar, SA- = soil agar without nutrient addition, SA+ = soil agar with nutrient addition, WYA = water yeast agar); error bars represent the standard error (n = 5). **Figure B. Relative abundance of the isolates on the different media.** Relative abundance of bacterial isolates grown on the different cultivation media averaged over the 5 replicates per medium (0.01TSA = 0.01 strength tryptone soy agar, 0.1TSA = 0.1 strength tryptone soy agar, DNB = dilute nutrient broth agar, SA- = soil agar without nutrient addition, SA+ = soil agar with nutrient addition, WYA = water yeast agar); error bars represent the standard error (n = 5). **Figure C. Cumulative growth on the different media.** Cumulative bacterial growth events over time on the different cultivation media (0.01TSA = 0.01 strength tryptone soy agar, 0.1TSA = 0.1 strength tryptone soy agar, DNB = dilute nutrient broth agar, SA- = soil agar without nutrient addition, SA+ = soil agar with nutrient addition, WYA = water yeast agar); each symbol represents one growth-event. **Figure D. Phylogenetic composition of isolates on the different media** Proportion of bacterial isolates belonging to the five different phylogenetic classes retrieved from the different cultivation media (0.01TSA = 0.01 strength tryptone soy agar, 0.1TSA = 0.1 strength tryptone soy agar, DNB = dilute nutrient broth agar, SA- = soil agar without nutrient addition, SA+ = soil agar with nutrient addition, WYA = water yeast agar).
(DOCX)

S1 Dataset Raw data. (XLSX)
