# Supplementary material for: Cultivation-success of rare soil bacteria is not influenced by incubation time and growth medium
Source: PLoS One. 2019 Jan 10;14(1):e0210073. doi: 10.1371/journal.pone.0210073 (PMC6328151; doi:10.1371/journal.pone.0210073)
Supplement: S1 File — Table A. Information on OTUs retrieved in this study. Unique OTUs, phylogenetic affiliation, relative abundance in the field soil and the number of retrieved isolates for each OTU in the present cultivation approach. Table B. Statistical results for differences between accumulation curves. Results of pairwise Kolmogorov-Smirnov tests between the accumulation curves of bacterial growth events on the different cultivation media (0.01TSA = 0.01 strength tryptone soy agar, 0.1TSA = 0.1 strength tryptone soy agar, DNB = dilute nutrient broth agar, SA- = soil agar without nutrient addition, SA+ = soil agar with nutrient addition, WYA = water yeast agar). Figure A. Cultivation success on the different media. Cultivation success (number of isolates relative to all 96 wells inoculated) of bacterial isolates on the different cultivation media (0.01TSA = 0.01 strength tryptone soy agar, 0.1TSA = 0.1 strength tryptone soy agar, DNB = dilute nutrient broth agar, SA- = soil agar without nutrient addition, SA+ = soil agar with nutrient addition, WYA = water yeast agar); error bars represent the standard error (n = 5). Figure B. Relative abundance of the isolates on the different media. Relative abundance of bacterial isolates grown on the different cultivation media averaged over the 5 replicates per medium (0.01TSA = 0.01 strength tryptone soy agar, 0.1TSA = 0.1 strength tryptone soy agar, DNB = dilute nutrient broth agar, SA- = soil agar without nutrient addition, SA+ = soil agar with nutrient addition, WYA = water yeast agar); error bars represent the standard error (n = 5). Figure C. Cumulative growth on the different media. Cumulative bacterial growth events over time on the different cultivation media (0.01TSA = 0.01 strength tryptone soy agar, 0.1TSA = 0.1 strength tryptone soy agar, DNB = dilute nutrient broth agar, SA- = soil agar without nutrient addition, SA+ = soil agar with nutrient addition, WYA = water yeast agar); each symbol represents one growth-event. Figure D. [file pone.0210073.s001.docx]

**S1 File**

**Table A S1 File**

| OTU number | Phylum | Class | Order | Family | Genus | Species | Relative abundance (%) | No. Isolates |
| --- | --- | --- | --- | --- | --- | --- | --- | --- |
| Otu000004 | Actinobacteria | Actinobacteria | Actinomycetales | Micrococcaceae | *Arthrobacter* | *psychrolactophilus* | 0.70986 | 1 |
| Otu000009 | Proteobacteria | Gammaproteobacteria | Pseudomonadales | Pseudomonadaceae | *Pseudomonas* | | 0.08773 | 8 |
| Otu000019 | Proteobacteria | Betaproteobacteria | Burkholderiales | Oxalobacteraceae | *Janthinobacterium* | | 0.20133 | 5 |
| Otu000043 | Proteobacteria | Betaproteobacteria | Burkholderiales | Comamonadaceae |  |  | 0.13353 | 2 |
| Otu000103 | Actinobacteria | Actinobacteria | Actinomycetales | Microbacteriaceae | *Microbacterium* | | 0.09286 | 1 |
| Otu000146 | Proteobacteria | Alphaproteobacteria | Caulobacterales | Caulobacteraceae | *Phenylobacterium* | | 0.08986 | 1 |
| Otu000168 | Actinobacteria | Actinobacteria | Kineosporiales | Kineosporiaceae | *Quadrisphaera* | | 0.10911 | 1 |
| Otu000182 | Firmicutes | Bacilli | Bacillales | Bacillaceae | *Bacillus* | *flexus* | 0.01102 | 1 |
| Otu000201 | Actinobacteria | Actinobacteria | Actinomycetales | Intrasporangiaceae | *Terracoccus* | | 0.06953 | 3 |
| Otu000323 | Actinobacteria | Actinobacteria | Actinomycetales | Nocardioidaceae | *Aeromicrobium* | | 0.02741 | 1 |
| Otu000410 | Proteobacteria | Alphaproteobacteria | Rhizobiales | Bradyrhizobiaceae | *Rhodopseudomonas* | | 0.01974 | 2 |
| Otu000486 | Proteobacteria | Alphaproteobacteria | Rhizobiales | Phyllobacteriaceae | *Mesorhizobium* | | 0.01251 | 10 |
| Otu000660 | Firmicutes | Bacilli | Bacillales | Paenibacillaceae | *Paenibacillus* | *amylolyticus* | 0.01559 | 1 |
| Otu001096 | Actinobacteria | Actinobacteria | Propionibacteriales | Nocardioidaceae | *Nocardioides* | | 0.00283 | 7 |
| Otu001144 | Proteobacteria | Gammaproteobacteria | Pseudomonadales | Pseudomonadaceae | *Pseudomonas* | *viridiflava* | 0.00000 | 20 |
| Otu001370 | Actinobacteria | Actinobacteria | Actinomycetales | Nocardioidaceae |  |  | 0.00116 | 1 |
| Otu001629 | Firmicutes | Bacilli | Bacillales | Staphylococcaceae | *Staphylococcus* | | 0.00434 | 1 |
| Otu002092 | Proteobacteria | Gammaproteobacteria | Enterobacteriales | Enterobacteriaceae | *Pantoea* |  | 0.00034 | 2 |
| Otu002494 | Proteobacteria | Alphaproteobacteria | Sphingomonadales | Sphingomonadaceae | *Sphingomonas* | | 0.00027 | 4 |
| Otu003445 | Proteobacteria | Alphaproteobacteria | Rhizobiales | Methylobacteriaceae | *Methylobacterium* | | 0.00027 | 1 |
| Otu004780 | Firmicutes | Bacilli | Lactobacillales | Carnobacteriaceae | *Carnobacterium* | | 0.00047 | 3 |
| Otu004967 | Proteobacteria | Betaproteobacteria | Burkholderiales | Oxalobacteraceae | *Janthinobacterium* | | 0.00000 | 3 |
| Otu005079 | Firmicutes | Bacilli | Bacillales | Staphylococcaceae | *Staphylococcus* | | 0.00027 | 5 |
| Otu005151 | Proteobacteria | Alphaproteobacteria | Rhizobiales | Xanthobacteraceae | *Ancylobacter* | | 0.00197 | 5 |
| Otu005676 | Proteobacteria | Alphaproteobacteria | Sphingomonadales | Sphingomonadaceae | *Sphingomonas* | *echinoides* | 0.00000 | 1 |
| Otu005905 | Proteobacteria | Alphaproteobacteria | Rhizobiales | Bradyrhizobiaceae | *Bradyrhizobium* | | 0.00034 | 1 |
| Otu007241 | Proteobacteria | Alphaproteobacteria | Sphingomonadales | Sphingomonadaceae | *Sphingomonas* | | 0.00000 | 1 |
| Otu007669 | Proteobacteria | Gammaproteobacteria | Pseudomonadales | Pseudomonadaceae | *Pseudomonas* | | 0.00000 | 1 |
| Otu008022 | Proteobacteria | Alphaproteobacteria | Rhizobiales | Bradyrhizobiaceae | *Bradyrhizobium* | | 0.00000 | 1 |
| Otu008409 | Proteobacteria | Betaproteobacteria | Burkholderiales | Burkholderiaceae | *Burkholderia* | | 0.00000 | 2 |
| Otu009814 | Proteobacteria | Gammaproteobacteria | Pseudomonadales | Pseudomonadaceae | *Pseudomonas* | | 0.00000 | 1 |
| Otu010816 | Proteobacteria | Betaproteobacteria | Burkholderiales | Oxalobacteraceae |  |  | 0.00000 | 1 |
| Otu012375 | Proteobacteria | Alphaproteobacteria | Rhizobiales | Phyllobacteriaceae | *Mesorhizobium* | | 0.00000 | 1 |
| Otu014999 | Proteobacteria | Betaproteobacteria | Burkholderiales | Comamonadaceae |  | *viridiflava* | 0.00000 | 2 |
| Otu015064 | Proteobacteria | Gammaproteobacteria | Pseudomonadales | Pseudomonadaceae | *Pseudomonas* | | 0.00000 | 1 |
| Otu015154 | Proteobacteria | Betaproteobacteria | Burkholderiales | Oxalobacteraceae | *Janthinobacterium* | *veronii* | 0.00000 | 1 |
| Otu015590 | Proteobacteria | Gammaproteobacteria | Pseudomonadales | Pseudomonadaceae | *Pseudomonas* | | 0.00000 | 1 |
| Otu016359 | Actinobacteria | Actinobacteria | Propionibacteriales | Propionibacteriaceae | *Microlunatus* | | 0.00000 | 3 |
| Otu016709 | Actinobacteria | Actinobacteria | Actinomycetales | Microbacteriaceae | *Cryocola* |  | 0.00000 | 1 |
| Otu017060 | Actinobacteria | Actinobacteria | Corynebacteriales | Nocardiaceae | *Rhodococcus* | | 0.00039 | 1 |
| Otu017699 | Proteobacteria | Betaproteobacteria | Burkholderiales | Oxalobacteraceae | *Janthinobacterium* | | 0.00047 | 1 |
| Otu018048 | Actinobacteria | Actinobacteria | Actinomycetales | Microbacteriaceae | *Microbacterium* | | 0.00000 | 2 |
| Otu021922 | Proteobacteria | Gammaproteobacteria | Xanthomonadales | Xanthomonadaceae | *Pseudoxanthomonas* | | 0.00000 | 1 |

**Table B S1 File**

| Medium | 0.01TSA | 0.1TSA | DNB | SA- | SA+ | WYA |
| --- | --- | --- | --- | --- | --- | --- |
| 0.01TSA |  | D=0.46  p=0.02* | D=0.34  p=0.23 | D=0.42  p=0.34 | D=0.26  p=0.58 | D=0.25  p=0.50 |
| 0.1TSA |  |  | D=0.64  p<0.01* | D=0.84  p<0.01* | D=0.46  p=0.08 | D=0.62  p<0.01* |
| DNB |  |  |  | D=0.62  p=0.09 | D=0.23  p=0.89 | D=0.49  p=0.05 |
| SA- |  |  |  |  | D=0.46  p=0.35 | D=0.22  p=0.98 |
| SA+ |  |  |  |  |  | D=0.47  p=0.07 |


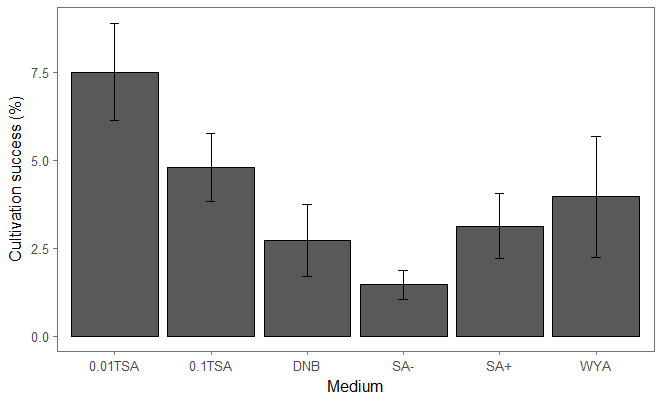


**Figure A S1 File**


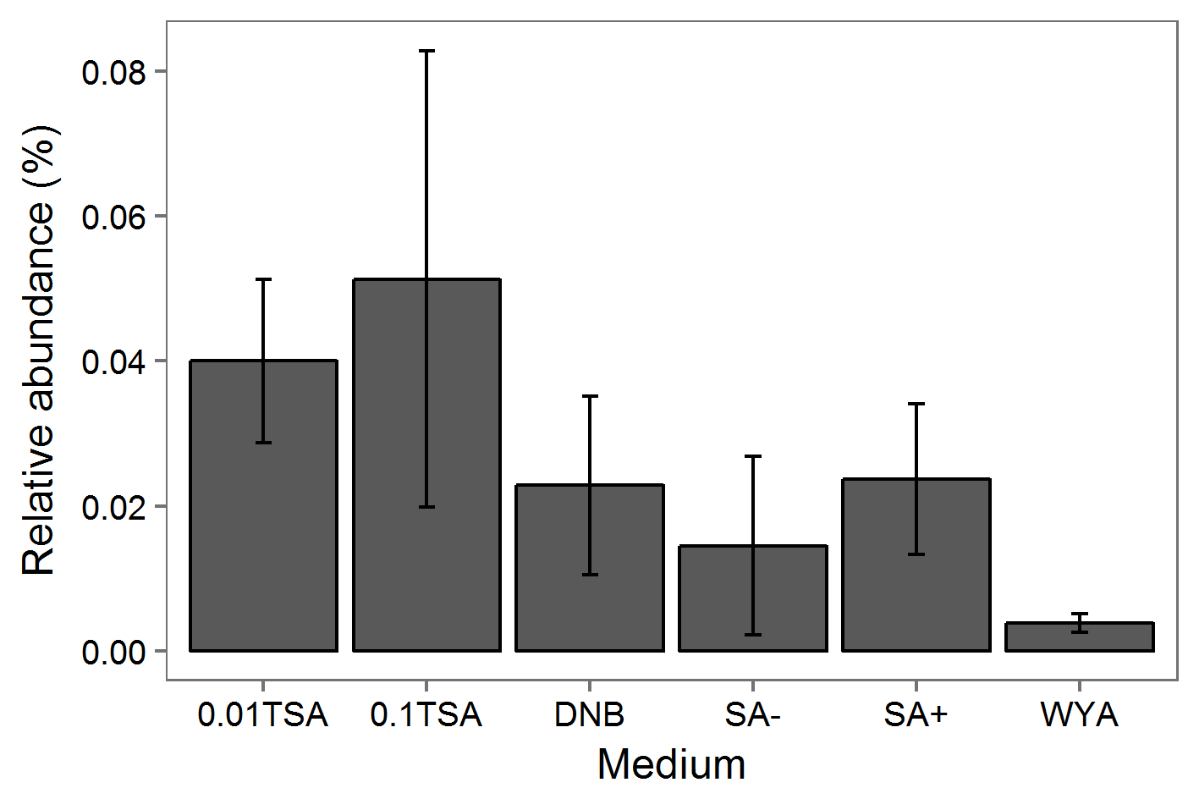


**Figure B S1 File**

**Figure C S1 File**


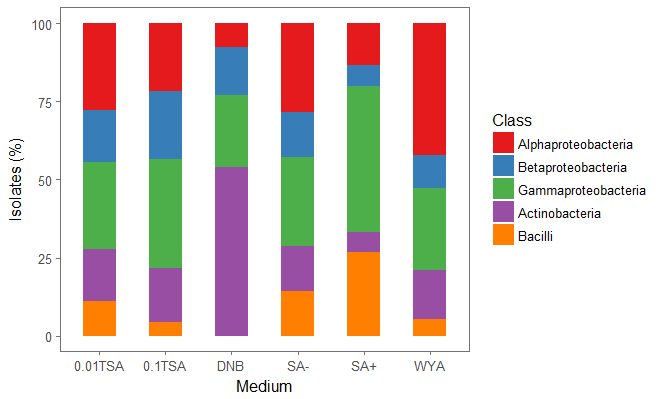


**Figure D S1 File**
